# Supplementary material for: The global response to the pandemic: An empirical cluster analysis of policies targeting COVID-19
Source: PLoS One. 2025 May 15;20(5):e0322692. doi: 10.1371/journal.pone.0322692 (PMC12080765; doi:10.1371/journal.pone.0322692)
Supplement: S1 Table — (DOCX) [file pone.0322692.s001.docx]

# Supplementary Table 1. Government response index

| **Containment and closure policies** | |
| --- | --- |
| C1 | School closings |
| C2 | Workplace closings |
| C3 | Cancel public events |
| C4 | Restrictions on gatherings |
| C5 | Close public transport |
| C6 | Stay at home requirement |
| C7 | Movement restrictions |
| C8 | International travel restrictions |
| **Economic policies** | |
| E1 | Income support |
| E2 | Debt relief |
| **Health system policies** | |
| H1 | Public information campaigns |
| H2 | Testing policy |
| H3 | Contact tracing |
| H6 | Facial coverings |
| H7 | Vaccination policy |
| H8 | Protection of elderly |
