# Supplementary material for: The global response to the pandemic: An empirical cluster analysis of policies targeting COVID-19
Source: PLoS One. 2025 May 15;20(5):e0322692. doi: 10.1371/journal.pone.0322692 (PMC12080765; doi:10.1371/journal.pone.0322692)
Supplement: S2 Table — (DOCX) [file pone.0322692.s002.docx]

# Supplementary Table 2. Countries included in the cluster analysis

| **Group A** | **Group B** | **Group C** | **Group D** |
| --- | --- | --- | --- |
| Albania | Andorra | Angola | Afghanistan |
| Algeria | Austria | Aruba | Belarus |
| Argentina | Bahrain | Benin | Burkina Faso |
| Australia | Belgium | Bolivia | Burundi |
| Azerbaijan | Bermuda | Bosnia and Herzegovina | Central African Republic |
| Bahamas | Bulgaria | Botswana | Nicaragua |
| Bangladesh | Croatia | Brunei | Niger |
| Barbados | Cyprus | Cameroon | Solomon Islands |
| Belize | Czech Republic | Chad | Sudan |
| Bhutan | Denmark | Congo | Tajikistan |
| Brazil | Estonia | Cote d'Ivoire | Tanzania |
| Cambodia | Faeroe Islands | Djibouti | Tonga |
| Canada | Finland | Dominica | Vanuatu |
| Cape Verde | France | Egypt | Yemen |
| Chile | Germany | El Salvador |  |
| China | Greece | Eritrea |  |
| Colombia | Greenland | Eswatini |  |
| Costa Rica | Hong Kong | Ethiopia |  |
| Cuba | Hungary | Gambia |  |
| Dominican Republic | Iceland | Ghana |  |
| Ecuador | Ireland | Guinea |  |
| Fiji | Israel | Haiti |  |
| Gabon | Japan | Jordan |  |
| Georgia | Laos | Lesotho |  |
| Guatemala | Latvia | Liberia |  |
| Guyana | Liechtenstein | Libya |  |
| Honduras | Lithuania | Madagascar |  |
| India | Luxembourg | Malawi |  |
| Indonesia | Macao | Mali |  |
| Iran | Malta | Mauritania |  |
| Iraq | Mauritius | Mozambique |  |
| Italy | Moldova | Namibia |  |
| Jamaica | Monaco | Nigeria |  |
| Kazakhstan | Netherlands | Papua New Guinea |  |
| Kenya | New Zealand | Paraguay |  |
| Kiribati | Norway | Senegal |  |
| Kuwait | Poland | Seychelles |  |
| Kyrgyz Republic | Portugal | Sierra Leone |  |
| Lebanon | Romania | Somalia |  |
| Malaysia | Russia | South Sudan |  |
| Mexico | San Marino | Syria |  |
| Mongolia | Saudi Arabia | Timor-Leste |  |
| Morocco | Serbia | Togo |  |
| Myanmar | Singapore | Turkmenistan |  |
| Nepal | Slovak Republic | United Arab Emirates |  |
| Oman | Slovenia | Uzbekistan |  |
| Pakistan | South Korea | Zambia |  |
| Palestine | Spain | Zimbabwe |  |
| Panama | Sweden |  |  |
| Peru | Switzerland |  |  |
| Philippines | United States Virgin Islands |  |  |
| Puerto Rico | Uruguay |  |  |
| Qatar |  |  |  |
| Rwanda |  |  |  |
| South Africa |  |  |  |
| Sri Lanka |  |  |  |
| Suriname |  |  |  |
| Thailand |  |  |  |
| Trinidad and Tobago |  |  |  |
| Tunisia |  |  |  |
| Turkey |  |  |  |
| Uganda |  |  |  |
| Ukraine |  |  |  |
| United Kingdom |  |  |  |
| United States |  |  |  |
| Venezuela |  |  |  |
| Vietnam |  |  |  |
