# Supplementary figures and images for: The global response to the pandemic: An empirical cluster analysis of policies targeting COVID-19
Source: PLoS One. 2025 May 15;20(5):e0322692. doi: 10.1371/journal.pone.0322692 (PMC12080765; doi:10.1371/journal.pone.0322692)

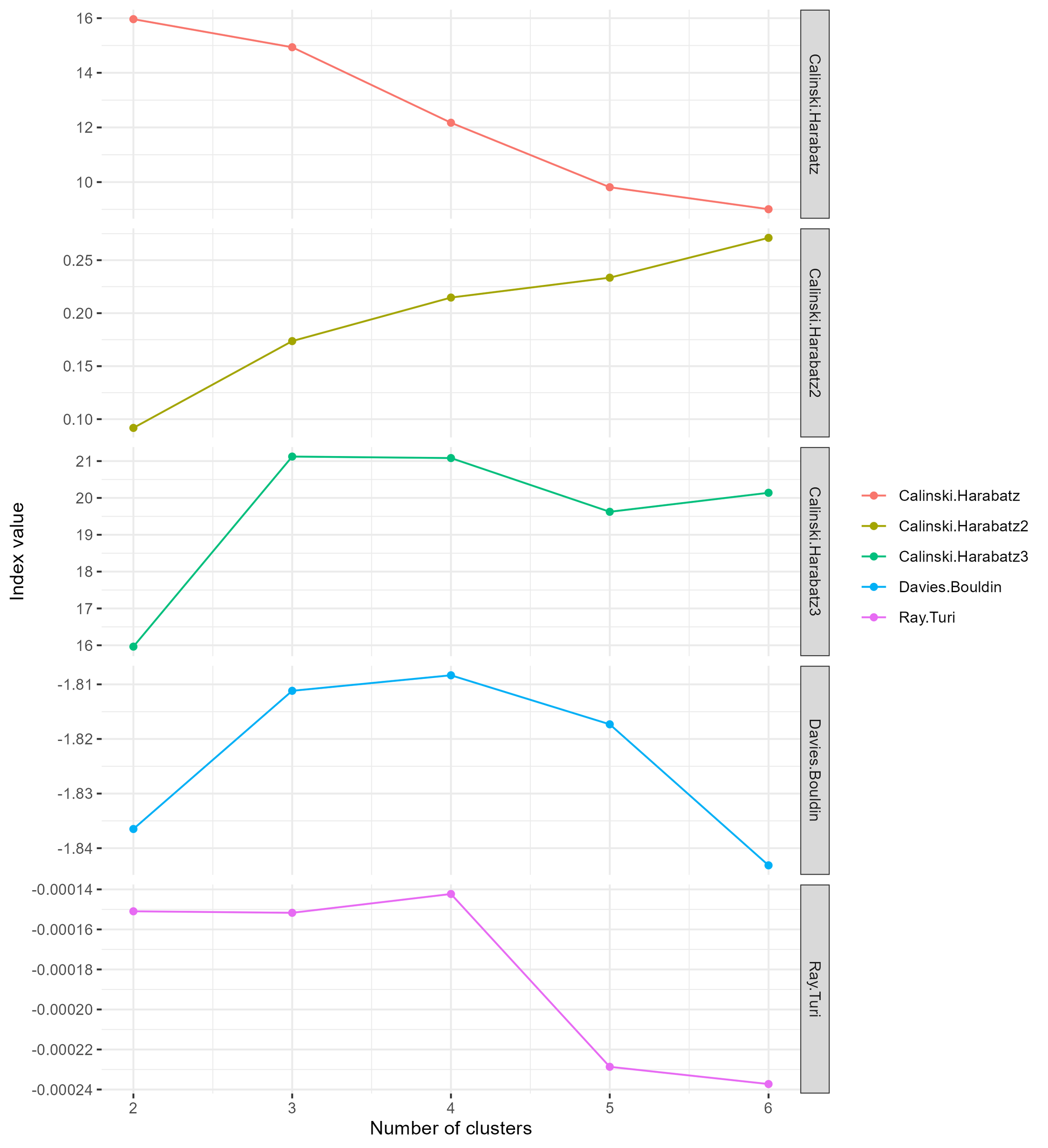

Supplement: S1 Fig — (TIF) [file pone.0322692.s003.tif]
