# Supplementary material for: The global response to the pandemic: An empirical cluster analysis of policies targeting COVID-19
Source: PLoS One. 2025 May 15;20(5):e0322692. doi: 10.1371/journal.pone.0322692 (PMC12080765; doi:10.1371/journal.pone.0322692)
Supplement: Attachment — Submitted filename: Response to reviewers.docx [file pone.0322692.s005.docx]

Revision of manuscript submitted to PLOS One

Title of manuscript: The global response to the pandemic: an empirical cluster analysis of policies targeting COVID-19

Manuscript ID: PONE-D-24-48926

# Editor comments

I found the study to have a potential to provide relevant contribution to the field. However, there are several areas where the manuscript could be improved or clarified to strengthen its impact:

**(E1) Methodology:** It would be beneficial to include a stronger rationale and justification for the use of a three-dimensional k-means clustering algorithm. Specifically, why this method was chosen over other clustering techniques, and how its application aligns with the study objectives, should be addressed more explicitly.

We appreciate the editor’s request for a stronger justification of our methodological approach. In response, we have expanded the rationale for using a three-dimensional k-means clustering algorithm in the methodology section. We selected a three-dimensional k-means clustering algorithm because it effectively captures multi-policy trajectories over time, clustering countries based on the type, intensity, and timing of interventions. This approach is well-suited for time-series data where multiple interrelated policy measures evolve dynamically. Alternative methods, such as principal component analysis combined with clustering, were considered but were not ideal for capturing both the multivariate structure and temporal evolution of policies. The 3D k-means method ensures that the cluster solution that emerges is data-driven, reflecting natural groupings of pandemic responses rather than imposing predefined number of states or Gaussian distributions in the data.

**(E2) Practical implications**: It would be good to clearly highlighting the practical implications of your research. Providing more explicit examples of how the findings could influence policy or practice would help bridge the gap between theoretical insights and real-world application.

Thank you! We have added a subsection to the discussion titled “Practical implications for pandemic response in the future”. Although it might be difficult to draw any straightforward epidemiological conclusions from our results (as discussed in the comment below), there are a number of issues to address.

**(E3) Results**: Consider exploring and discussing whether the observed differences in strategies are associated with better or worse performance in terms of epidemiological criteria. Identifying and analyzing such patterns could significantly enhance the utility and applicability of the findings.

This is a very important comment and reflects one of our initial ambitions with this paper. However, for a number of reasons that became clear in the course of the analysis, the link to epidemiological criteria is difficult to make. The most straightforward reason being the within-heterogeneity of the very large clusters (e.g., cluster A includes approximately 73 percent of the global population). We have provided further rationale for this decision in the section “methodological considerations”.

**(E4) Figure quality**: The quality of the figures should be improved for better readability and visual impact. Ensure that all figures are clear, professional, and effectively convey the intended information.

We have redrawn the figures and hope that they are more readable and they have improved in regards to conveying the intended information. However, we can easily amend them if the editors have specific suggestions of how to further improve their quality.

**(E5) Rationale for dimensions**: Provide a more detailed rationale for selecting type, intensity, and timing as your dimensions of analysis. How do these dimensions align with or advance the existing literature? Discuss whether other potential domains, such as scope (geography, demography), enforcement, communication, compliance, equity, or resource intensity, were considered and justify their exclusion if relevant.

Thank you for this comment. We think the chosen dimensions (type, intensity and timing) exhaust the possibilities provided by the OxCGRT measure and we have not found any alternative data sources with this type of granular data for such a large number of countries. The fact that we were not able to consider these other important dimensions is however a clear limitation of the paper. We have included a sentence on recommendations for future research paying particular consideration to these domains.

# Reviewer #1

Please use the space provided to explain your answers to the questions above. You may also include additional comments for the author, including concerns about dual publication, research ethics, or publication ethics. (Please upload your review as an attachment if it exceeds 20,000 characters)

Reviewer #1: Understanding the trajectories of responses to COVID-19 is an interesting and important topic for research (it is, in fact, a question that I have spent some time thinking about too) and this paper takes a stab at it. However, I have some concerns about the statistical approach and assumptions that make me question how things were done.

**(R1_1)** In particular, I think there is a hidden assumption in their approach that limits the usefulness of 3d k-means clustering. My issue can best be seen by the increases one observes in stringency in late 2021 and early 2022 when Omicron was first spreading. Essentially, these policies can be or are dynamic and respond to changes in the incidence of disease. As a result, the clustering algorithm ALSO imposes a uniformity of incidence patterns across clusters. There are alternatives that the authors could take, principally by introducing tools like dynamic time warping that allow for differences in the timing (but not the sequence) of interventions. I would like to see the authors take such an approach to generate clusters that are less reliant on the precise timing of changes in policies, but rather relies on the sequence of policies.

We thank the reviewer for this helpful suggestion. We have adjusted our analysis to better account for the dynamic nature of policy responses. Instead of relying on a fixed calendar timeline, we now allow for differences in the timing of interventions by anchoring policy trajectories relative to each country’s first registered COVID-19 case. This adjustment ensures that countries are compared based on their response progression rather than absolute dates, mitigating the issue of arbitrary temporal alignment. Despite this adjustment, clustering may still be biased by underlying incidence patterns. Variations in testing policies and deficiencies in global epidemiological data (e.g., excess mortality) prevented us from accounting for this. This is now addressed in the methodological considerations

We acknowledge that dynamic time warping (DTW) is a useful technique for handling time-series data with temporal shifts. However, after careful consideration, we did not think it would be commensurable with the multiple trajectories that we are clustering, rather it seemed useful when comparing two trajectories that can be temporally matched. It is therefore of limited use in multi-dimensional multi-trajectory analysis because it is originally a pairwise method. We are aware that it is possible to compute an average trajectory and align all the trajectories with this, but given the high dimensionality the comparisons become computationally expensive and the average trajectory lacks a natural interpretation.

**(R1_2)** I am also concerned that the choice to present four clusters is arbitrary and the authors have provided no justification for doing so. Typically one would present a skree plot or some similar metrics to justify a particular number of clusters but I could not find any such plot in the paper or supplemental materials. As a result, I am unsure what to make of the results. This issue is exacerbated when I look at the country map and there are things that don't really pass the smell test. How can New Zealand, for example, be in the same class as Sweden? Two countries that exhibited very different policy responses on a number of margins.

We thank the reviewer for raising these concerns. We acknowledge the importance of justifying the number of clusters and clarifying the logic behind the resulting groupings.

To address the first point, we selected four clusters based on a number of criteria available in the klm3d package, including Calinski-Harabasz index, two variations on this (Kryszczuk and Gendolini), and the Davies–Bouldin and Ray-Turi indices. Essentially all criteria attempt to balances within-cluster variance minimization and between-cluster separation. To clarify this decision, we have now included a plot of all five diagnostic criteria in the supplemental materials, comparing 2,3, 4, 5, and 6 cluster solutions and found that while they produced slightly different configurations, the four-cluster solution was the optimal number for both the Davies–Bouldin and Ray-Turi indices and very similar to the 3 cluster solution for Calinski-Harabasz (Gendolini) index. We hope these plots will convince the reviewer that the 4 cluster solution is optimal.

Regarding the second concern, we recognize that certain country pairings may seem unexpected. However, the clustering algorithm captures the overall trajectory of pandemic responses across multiple policy dimensions and over nearly three years, rather than focusing on individual policies at certain times in isolation. While Sweden and New Zealand differed in specific policy choices, their overall response patterns—including the intensity and timing of interventions—shared enough similarities to be grouped together within the identified framework. We have now expanded the discussion in the manuscript to clarify this point.

**(R1_3)** - I would love to see this analysis broken into two steps, rather than the current one step approach. First, cluster the daily profile of policies (and possibly present the results in a heat map). Then use dynamic time warping and 3d k-means clustering to group the daily policy clusters into the trajectory clusters.

Thank you. The rationale behind our chosen methodology is that pandemic responses were multifaceted, dynamic and evolving, making it crucial to capture the full trajectory of policy changes rather than individual daily snapshots. Clustering daily policy profiles separately would segment policies at a specific moment in time, whereas our 3D k-means clustering approach is designed to identify long-term strategic patterns across multiple policy domains.

However, we appreciate the reviewer’s comments, as they prompted reflection on the alternative methods we explored during data analysis. One approach we initially considered was performing a principal component analysis (PCA) on daily policy data, extracting the first few principal components, and then clustering those components. The goal was to identify dominant country contributions to each eigenvector. However, the policies did not correlate strongly, resulting in low explained variance—the first principal component accounted for only 25% of the variation, while the second explained 18%. Furthermore, no clear pattern emerged in which countries contributed predominantly to a single component. Given these limitations, we ultimately abandoned this approach, as it proved impractical and did not meaningfully group policy strategies. We also explored heatmaps to visualize correlations between policy measures, which provided interesting insights into relationships between interventions over time. However, given the sheer number of time points (over 1,000 daily observations), the volume of heatmaps was too large to effectively summarize the data in a meaningful way.

(R1_4) - As a final side note--there were changes in political leadership across countries during this time period that may have affected the policy choices, but again there is an assumption that these changes in leadership do not affect the trajectory cluster.

We appreciate the reviewer’s observation regarding political leadership changes and their potential impact on pandemic policy trajectories. However, we would like to clarify that our clustering approach does not assume that leadership changes have no effect on pandemic responses. Rather, the method captures the overall trajectory of policies as they evolved over time, regardless of whether shifts were driven by leadership changes, epidemiological factors, or other political and socioeconomic dynamics.

That said, we acknowledge that leadership transitions in certain countries (such as the United States) were associated with significant shifts in pandemic policies. We have now expanded the section on methodological considerations to briefly address this point, recognising that political transitions may have influenced policy trajectories in some cases. However, our results suggest that broader structural factors, such as welfare state arrangements and economic resilience, also played a major role in shaping national response strategies beyond individual leadership changes.

# Reviewer #2

Method session

**(R2_1)** It is unclear to the reviewer how countries were clustered into the four strategy groups. Were the 3D k-means used for clustering policy strategies that simultaneously clustered county groups?

Thank you for this comment! To clarify, the 3D k-means clustering algorithm was applied directly to the policy trajectories, meaning that countries were not grouped manually or separately but rather assigned to clusters based on the similarity of their policy response trajectories across the three dimensions (type, intensity, and timing). The algorithm partitions these multivariate time-series trajectories into clusters, where the within-cluster variance is minimized.

Thus, the four strategy groups emerged entirely from the clustering of policy trajectories – countries that followed similar response patterns over time were grouped together. The resulting clusters, which are entirely data driven, represent distinct overarching approaches to pandemic management, rather than predefined country groups.

We recognize the need for additional clarity on this point and have revised the methodology section to explicitly state that the clustering was performed on policy trajectories, leading to the identified country groups.

**(R2_2)** The authors used a novel approach for grouping countries using 3D k-means clustering based on the policy strategies. As policies throughout COVID change frequently in each country, have the authors considered re-clustering the country groups based on different times of COVID? The clusters might vary at the beginning of COVID compared to Omicron and late COVID time.

We appreciate the reviewer’s comments regarding the timing of measurement. As noted in response to Reviewer 1, we have adjusted the alignment of policy trajectories to begin relative to each country’s first registered COVID-19 case, rather than a fixed calendar date. This ensures that countries are compared at comparable points in their pandemic response, reducing distortions due to variation in outbreak timing.

We considered looking at different waves or phases of the pandemic but quickly realized that on a global scale, these phases were asynchronous and would be very difficult to determine in an objective and systematic way. The reported cases per capita reflects testing capacity as well as epidemiologic spread and therefore cannot be relied on for methodological analyses.

**(R2_3)** Were there any missing values in the data? If so, how were missing values being handled?

Yes, there was missing values in the data. A total of 9 countries (Comoros, Kiribati, Solomon Islands, Tonga, Vanuatu, Turkmenistan, Cape Verde, Luxembourg, Trinidad and Tobago) all with small populations was excluded from the data either because of missing data or because the first reported case happened later on in the pandemic. This is now mentioned in the materials and methods section.

**(R2_4)** Was 4 clusters the optimal number of clusters identified by the Calinski-Habaratz index?

The selection of four clusters was based on a careful inspection of a number of cluster criteria, including the Calinski-Habaratz index. We have now clarified this point in the methodology section.

**(R2_5)** Have the authors considered running a sensitivities analysis on different numbers of clutters and comparing the temporal patterns of the government response index?

Thank you. As described in the methodology, we tested multiple cluster solutions (ranging from 2 to 6 clusters) and selected the four-cluster solution based on five separate cluster criteria indices, which provided the best balance between within-cluster cohesion and between-cluster separation. This process inherently involved a sensitivity analysis of different cluster numbers.

Results session

**(R2_1)** Figure 1: It wasn’t clear to the reviewer what the y-axis “index_mean” means. Please clarify.

The ‘index_mean’ represents the average policy stringency level for each cluster over time, calculated based on the normalized values of the OxCGRT policy indicators. Each line corresponds to the mean value of a given policy measure within a cluster, allowing for a direct comparison of how different strategy groups implemented and adjusted their policies over the course of the pandemic. However, we appreciate that this may have been a bit confusing and have renamed this to “mean response value”.

To improve clarity, we have updated the figure caption to explicitly define ‘index_mean’, and we have revised the manuscript text where Figure 1 is referenced to ensure this is clearly communicated.

**(R2_7)** The authors stated that “the majority of countries adopting the second strategy B.. . Based on the supplementary material, it seems that group A had the highest number of countries.

We have reworded this sentence in the manuscript to make it clear that we are describing the dominant characteristics of countries within Strategy B, rather than the relative size of the cluster compared to others.

**(R2_8)** - Table 1: Could you please provide more information regarding the clustering of the strategies? Of the 16 government response indexes, which are the ones for groups A, B, C, and D? Would you be able to generate a summary statistic of the total number of types of interventions and the mean and SD for the timing and duration of interventions, please?

To clarify, the clustering algorithm was applied simultaneously to all 16 government response measures, rather than selecting or weighting specific interventions. This means that countries were grouped based on the full trajectory of all 16 policies over time, capturing both intensity and timing in a multidimensional space. We have added a sentence about this in the methods section of the paper.

**(R2_9)** - Figure 3: The authors might consider a violin plot for trying to show the distribution of each group and facet by demographic, economic and political profile. It is unclear to the reviewer what the x axis “scaled values mean. Also, figure 3 was not mentioned in the results.

We appreciate the reviewer’s suggestion to use a violin plot. While violin plots are valuable for visualizing density distributions, we chose to use box plots with jittered points to clearly display the range, central tendency, and outliers of each indicator while maintaining direct comparability across clusters. Given the variation in cluster sizes—where Cluster D includes only 14 countries compared to 67 in Cluster A—violin plots could create misleading impressions of distribution density. We believe that the current visualization provides the best balance between readability and accuracy. However, we have refined the figure’s presentation to enhance clarity. E.g., we have renamed the x axis and added a caption to the table. We have also corrected the typo referring to “figure 2 instead of “figure 3, thank you for pointing this out.

Discussion session

10 - The authors may consider referencing: Lyu S, Qian C, McIntyre A, Lee CH. One Pandemic, Two Solutions: Comparing the U.S.-China Response and Health Priorities to COVID-19 from the Perspective of Two Types of Control. Healthcare (Basel). 2023 Jun 26;11(13):1848. doi: 10.3390/healthcare11131848. PMID: 37444682; PMCID: PMC10341116.

Thank you! We will make a reference to this article when acknowledging the importance of political culture and leadership for the pandemic response.

# Journal Requirements:

When submitting your revision, we need you to address these additional requirements.

1. Please ensure that your manuscript meets PLOS ONE's style requirements, including those for file naming. The PLOS ONE style templates can be found at

<https://journals.plos.org/plosone/s/file?id=wjVg/PLOSOne_formatting_sample_main_body.pdf> and

[https://journals.plos.org/plosone/s/file?id=ba62/PLOSOne_formatting_sample_title_authors_affiliations.pdf](https://track.editorialmanager.com/CL0/https:%2F%2Fjournals.plos.org%2Fplosone%2Fs%2Ffile%3Fid=ba62%2FPLOSOne_formatting_sample_title_authors_affiliations.pdf/1/010f019489630ae8-f2c3d0ca-4521-4527-8db3-ccbadb97cd4b-000000/RgTKf5ayra0WBos3XxABywU8IkaKh8VK2g9ph3kv0Ys=194)

Thank you. We have revised the manuscript according to the PLOS ONE style requirements.

2. Please note that PLOS ONE has specific guidelines on code sharing for submissions in which author-generated code underpins the findings in the manuscript. In these cases, we expect all author-generated code to be made available without restrictions upon publication of the work. Please review our guidelines at <https://journals.plos.org/plosone/s/materials-and-software-sharing#loc-sharing-code> and ensure that your code is shared in a way that follows best practice and facilitates reproducibility and reuse.

OK!

3. Thank you for stating the following financial disclosure:

 “Funding for the work on this study was provided by the Swedish Research Council for Health, Working Life and Welfare (project no. 2022 00262)

Please state what role the funders took in the study.  If the funders had no role, please state: The funders had no role in study design, data collection and analysis, decision to publish, or preparation of the manuscript.

If this statement is not correct you must amend it as needed.

Please include this amended Role of Funder statement in your cover letter; we will change the online submission form on your behalf.

Thank you. We have included this statement in the cover letter

4. Thank you for stating the following in the Acknowledgments Section of your manuscript:

“Funding for the work on this study was provided by the Swedish Research Council for Health, Working Life and Welfare (project no. 2022 00262)

We note that you have provided funding information that is not currently declared in your Funding Statement. However, funding information should not appear in the Acknowledgments section or other areas of your manuscript. We will only publish funding information present in the Funding Statement section of the online submission form.

Please remove any funding-related text from the manuscript and let us know how you would like to update your Funding Statement. Currently, your Funding Statement reads as follows:

“Funding for the work on this study was provided by the Swedish Research Council for Health, Working Life and Welfare (project no. 2022 00262)

Please include your amended statements within your cover letter; we will change the online submission form on your behalf.

Thank you. We have removed the funding information from the acknowledgement section and would like to include this information in the funding statement instead.

5. We note that [Figure 2] in your submission contain [map/satellite] images which may be copyrighted. All PLOS content is published under the Creative Commons Attribution License (CC BY 4.0), which means that the manuscript, images, and Supporting Information files will be freely available online, and any third party is permitted to access, download, copy, distribute, and use these materials in any way, even commercially, with proper attribution. For these reasons, we cannot publish previously copyrighted maps or satellite images created using proprietary data, such as Google software (Google Maps, Street View, and Earth). For more information, see our copyright guidelines: <http://journals.plos.org/plosone/s/licenses-and-copyright>.

We require you to either (1) present written permission from the copyright holder to publish these figures specifically under the CC BY 4.0 license, or (2) remove the figures from your submission:

a. You may seek permission from the original copyright holder of Figure 2 to publish the content specifically under the CC BY 4.0 license.

We recommend that you contact the original copyright holder with the Content Permission Form (<http://journals.plos.org/plosone/s/file?id=7c09/content-permission-form.pdf>) and the following text:

“I request permission for the open-access journal PLOS ONE to publish XXX under the Creative Commons Attribution License (CCAL) CC BY 4.0 (<http://creativecommons.org/licenses/by/4.0/>). Please be aware that this license allows unrestricted use and distribution, even commercially, by third parties. Please reply and provide explicit written permission to publish XXX under a CC BY license and complete the attached form.

Please upload the completed Content Permission Form or other proof of granted permissions as an Other file with your submission.

In the figure caption of the copyrighted figure, please include the following text: “Reprinted from [ref] under a CC BY license, with permission from [name of publisher], original copyright [original copyright year].

b. If you are unable to obtain permission from the original copyright holder to publish these figures under the CC BY 4.0 license or if the copyright holder’s requirements are incompatible with the CC BY 4.0 license, please either i) remove the figure or ii) supply a replacement figure that complies with the CC BY 4.0 license. Please check copyright information on all replacement figures and update the figure caption with source information. If applicable, please specify in the figure caption text when a figure is similar but not identical to the original image and is therefore for illustrative purposes only.

The following resources for replacing copyrighted map figures may be helpful:

USGS National Map Viewer (public domain): <http://viewer.nationalmap.gov/viewer/>

The Gateway to Astronaut Photography of Earth (public domain): <http://eol.jsc.nasa.gov/sseop/clickmap/>

Maps at the CIA (public domain): <https://www.cia.gov/library/publications/the-world-factbook/index.html> and <https://www.cia.gov/library/publications/cia-maps-publications/index.html>

NASA Earth Observatory (public domain): <http://earthobservatory.nasa.gov/>

Landsat: <http://landsat.visibleearth.nasa.gov/>

USGS EROS (Earth Resources Observatory and Science (EROS) Center) (public domain): [http://eros.usgs.gov/#](http://eros.usgs.gov/)

Natural Earth (public domain): [http://www.naturalearthdata.com/](http://track.editorialmanager.com/CL0/http:%2F%2Fwww.naturalearthdata.com%2F/1/010f019489630ae8-f2c3d0ca-4521-4527-8db3-ccbadb97cd4b-000000/bT23spq6VV54oZlrpS44veBx3kO0LpOr-ogev77b0v4=194)

We have now included an acknowledgement to the website used to generate the map following their guidelines for licencing: [Terms of Use | MapChart](https://www.mapchart.net/terms.html#licensing-maps)

6. Please include a copy of Table 2 which you refer to in your text on page 6.

Thank you for noticing this mistake. We meant to refer to Table 1 and have revised the text.

7. We note you have included a table to which you do not refer in the text of your manuscript. Please ensure that you refer to Table 1 in your text; if accepted, production will need this reference to link the reader to the Table.

See above

8. Please include captions for your Supporting Information files at the end of your manuscript, and update any in-text citations to match accordingly. Please see our Supporting Information guidelines for more information: [http://journals.plos.org/plosone/s/supporting-information](http://track.editorialmanager.com/CL0/http:%2F%2Fjournals.plos.org%2Fplosone%2Fs%2Fsupporting-information/1/010f019489630ae8-f2c3d0ca-4521-4527-8db3-ccbadb97cd4b-000000/pObUOkSEmWUQI4-es1OYQBSwsvadKXthZSFOGG0I1Pw=194).

OK!
